# Cephalometric measurements performed on CBCT and reconstructed lateral cephalograms: a cross-sectional study providing a quantitative approach of differences and bias

**DOI:** 10.1186/s12903-022-02131-3

**Published:** 2022-03-30

**Authors:** Benedetta Baldini, Davide Cavagnetto, Giuseppe Baselli, Chiarella Sforza, Gianluca Martino Tartaglia

**Affiliations:** 1 Facial Surgery and Dentistry Fondazione IRCCS Cà Granda, UOC Maxillo, Ospedale Maggiore Policlinico, 20142 Milan, Italy; 2 Department of Oral and Maxillofacial Surgery, Amsterdam University Medical Center (Amsterdam UMC), Location AMC, Meibergdreef 9, 1105 AZ Amsterdam, The Netherlands; 3 Department of Surgical Sciences, University of Torino, Via Nizza 230, 10126 Torino, Italy; 4 Department of Electronics, Information and Bioengineering, Politecnico Di Milano, 20133 Milano, Italy; 5 Faculty of Medicine and Surgery, Department of Biomedical Sciences for Health, Functional Anatomy Research Center (FARC), Università Degli Studi Di Milano, Milan, Italy; 6 Department of Biomedical, Surgical and Dental Sciences, School of Dentistry, University of Milan, 20100 Milan, Italy

**Keywords:** Cephalometric analysis, CBCT, Three-dimensional imaging, Orthodontics, Maxillofacial, Bland–Altman analysis, Agreement

## Abstract

**Background:**

Cephalometric analysis is traditionally performed on skull lateral teleradiographs for orthodontic diagnosis and treatment planning. However, the skull flattened over a 2D film presents projection distortions and superimpositions to various extents depending on landmarks relative position. When a CBCT scan is indicated for mixed reasons, cephalometric assessments can be performed directly on CBCT scans with a distortion free procedure. The aim of the present study is to compare these two methods for orthodontic cephalometry.

**Methods:**

114 CBCTs were selected, reconstructed lateral cephalometries were obtained by lateral radiographic projection of the entire volume from the right and left sides. 2D and 3D cephalometric tracings were performed. Since paired t-tests between left and right-side measurements found no statistically significant differences, mean values between sides were considered for both 2D and 3D values. The following measurements were evaluated: PNS-A; S-N; N-Me; N-ANS; ANS-Me; Go-Me; Go-S; Go-Co; SNA, SNB, ANB; BaŜN; S-N^PNS-ANS; PNS-ANS^Go-Me; S-N^Go-Me. Intraclass correlation coefficients, paired t-test, correlation coefficient and Bland–Altman analysis were performed to compare these techniques.

**Results:**

The values of intra- and inter-rater ICC showed excellent repeatability and reliability: the average (± SD) intraobserver ICCs were 0.98 (± 0.01) and 0.97(± 0.01) for CBCT and RLCs, respectively; Inter-rater reliability resulted in an average ICC (± SD) of 0.98 (± 0.01) for CBCT and 0.94 (± 0.03) for RLC. The paired t-tests between CBCT and reconstructed lateral cephalograms revealed that Go-Me, Go-S, PNS-ANS^Go-Me and S-N^Go-Me measurements were statistically different between the two modalities. All the evaluated sets of measurements showed strong positive correlation; the bias and ranges for the 95% Limits of Agreement showed higher levels of agreement between the two modalities for unpaired measurements with respect to bilateral ones.

**Conclusion:**

The cephalometric measurements laying on the mid-sagittal plane can be evaluated on CBCT and used for orthodontic diagnosis as they do not show statistically significant differences with those measured on 2D lateral cephalograms. For measurements that are not in the mid-sagittal plane, the future development of specific algorithms for distortion correction could help clinicians deduct all the information needed for orthodontic diagnosis from the CBCT scan.

## Background

Cephalometric analysis was defined by Moyers as a crucial tool to improve our understanding of the morphological characteristics of craniomaxillary structures [1]. It represents the reference standard for evaluating the bony structures for orthodontic purposes in conjunction with orthopantomography [1]. However, two-dimensional (2D) lateral cephalometric analyses is hindered by several limitations when a three-dimensional (3D) object is to be studied, because a 3D anatomical structure flattened on a sagittal plane presents distortions and superimposition of bony structures [2]. Projection errors depend on the representation of a 3D object on a 2D image and to the imaging technology, in particular the distance between the focus, the head, and the film. The resulting superimposition of anatomical structures complicates image interpretation and landmark identification. These distortions reduce measurement accuracy, especially for landmarks far from the midsagittal plane (MSP) [3].

The use of multi-slice computed tomography (MSCT), allowed to get rid of these limitations and to perform a 3D evaluation of bony structures thus solving the problem of overlapped reference areas [3]. On the other hand, this imaging modality delivers a significantly higher dose of ionizing radiation compared to 2D cephalograms and therefore its application was restricted to specific cases [4–6]. Recently, the development of cone-beam computed tomography (CBCT) allowed to obtain precise 3D imaging with consistently less radiation compared to conventional CT scans [7–10]. However, since it delivers a significantly higher radiation dose compared to lateral teleradiography, the latter remains the method of choice to assess malocclusions and maxillofacial growth; and to evaluate the effect of facial orthopaedics, orthodontics and orthognathic surgery [11, 12].

The limitation of conventional cephalometric measurements and their potential impact on orthodontic diagnosis was assessed in some studies focused on the direct comparison between CBCT and lateral cephalograms [13, 14]. However, the need to obtain both imaging modalities in each patient made it more complex to collect samples large enough to draw meaningful conclusions. Since Kumar et al. [15] demonstrated no significant differences between linear distances and angles assessed with reconstructed lateral cephalometric radiographs (RLC a 2D image that can be obtained from a CBCT scan), and lateral skull teleradiography, it is possible to obtain 2D images from CBCT scans taken for specific indications and use them for research purposes.

The papers by the research groups leaded respectively by Baumrind and Frantz [11, 12] on the reliability of 2D landmarks reported the envelopes of error for cephalometric points on skull lateral teleradiography. Considering the ease in landmark identification and the lack of distortion, the envelope of error of CBCT cephalometry is expected to be different both in shape and magnitude [14]. Available literature on 3D landmark reliability, according to the authors’ scoping review, demonstrated a greater reliability for many cephalometric measurements than the one performed on 2D cephalometric analysis [16–18].

However, the specific envelopes of error of 3D cephalometric points have not been investigated thoroughly and, even more important, we still miss the correspondent set of reference values. The primary aim of this paper is to measure the differences between 2 and 3D cephalometric variables, focusing on the differences between angles and distances coplanar to the mid-sagittal plane and those that lay on a different plane and are therefore more subject to errors. To the scope, the hypothesis that measurements not belonging to the MSP are affected by a mixed amount of distortion depending on their 3D position will be tested. The data gathered in this study can be a valid starting point for the further analysis of correction coefficients for 3D comparison with 2D normal values of the different measurements depending on the distribution of the errors provided by the Bland–Altman analysis.

## Materials and methods

### Study design and sample selection

The patients’ records used in the present cross-sectional study were retrieved from the Dental Department of the Fondazione IRCCS Cà Granda Ospedale Maggiore Policlinico, Milan, Italy. The inclusion criteria were:

1. All subjects presenting full permanent dentition apart from the third molar;
2. Skeletal Class I according to Steiner (ANB angle between 0° and 4°, measured on the latero-lateral projection) and a maximum difference of 3 mm between the distance of each Gonion and Maxillaris point from the MSP in the postero-anterior projection [19];
3. Absence of cross-bite as reported in patients’ records and confirmed on the CBCT scans.

The exclusion criteria were:

1. Missing molars or premolars;
2. Previous orthodontic treatment;
3. Altered bone metabolism;
4. Skeletal asymmetry between right and left cephalometric variables greater than 2 mm;
5. Alterations to the maxillofacial skeleton (acquired or congenital).

The purpose of selecting symmetric skeletal Class I subjects is to design a simplified model in which the difference between imaging modalities was as much as possible due to the measuring instruments and not to the differences between patients [14].

A total of 750 CBCT taken from January 2012 to June 2016 for mixed reasons (impacted and supernumerary teeth; bicuspid tooth implant needs; obstructive sleep disorders breathing and apnea syndrome; orthognathic surgery; trauma not involving mandibular or maxillary position; foreign objects) at the Dental Department of the Fondazione IRCCS Cà Granda Ospedale Maggiore Policlinico were reviewed and 114 patients’ CBCTs were selected. The sample was composed of Caucasian subjects: 56 males aged between 7 and 21 years, and 59 females between 8 and 19.5 years. All the CBCTs were performed with the same scanner, I-CAT FLX (Imaging Sciences International, Hatfield, PA, USA). The scanning protocol involved a 4 mm slice thickness, a 16 × 22 cm field of view, a 20-s scan time, and a 0.49/0.49/0.5 mm voxel size.

### Ethical approval and informed consent

The ethical approval of the present study was obtained by the Fondazione IRCCS Cà Granda, Ospedale Maggiore, Milan, Italy (09/03/2016; n. 421). The protocol was designed in accordance with the Helsinki Declaration, including all amendments and revisions. All patients and the appropriated person who has parental authority gave their written informed consent for all the procedures that are described hereafter and for the data gathered from their records to be used for scientific purposes.

### Data elaboration

**Fig. 1:**
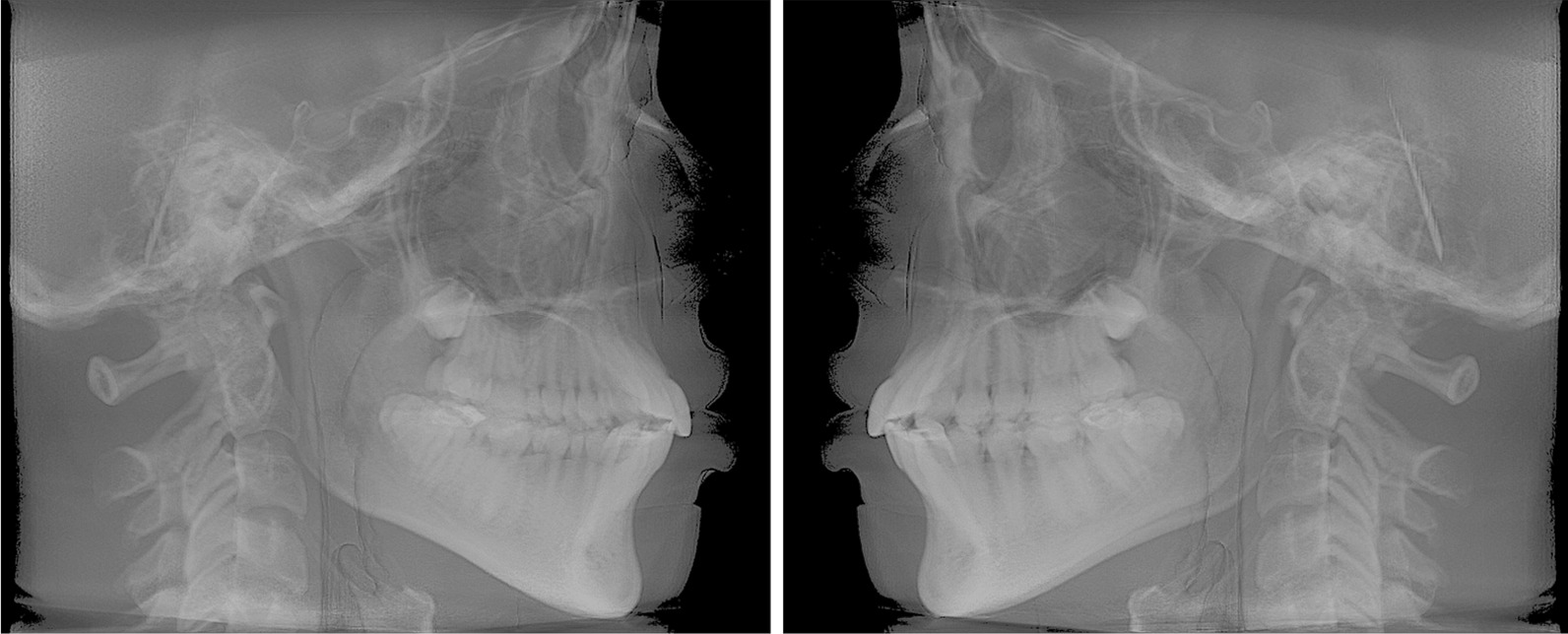
Right and left reconstructed lateral cephalograms

Raw data from the CBCT scan were coded into Digital Imaging and Communications in Medicine (Dicom3) file format. These data were then processed into Mimics software to perform 3D cephalometric tracings (version 20.0, Materialise, Leuven, Belgium; https://www.materialise.com/en/medical/mimics-innovation-suite/mimics). Two RLCs (right and left) were reconstructed for each CBCT scan by lateral radiographic projection of the entire volume using iCAT Vision software (Imaging Sciences International, Inc., https://ct-dent.co.uk/i-cat-vision/) (Fig. 1). All 2D cephalograms were then traced using a dedicated software (Dolphin Imaging Cephalometric and Tracing Software, version 11.9, Chatsworth, California, https://www.dolphinimaging.com/Media/DolphinNews?Subcategory_OS_Safe_Name=20160913).

Cephalometric points on CBCT scans were firstly identified in one plane (axial, coronal or sagittal) and then checked in the other two and in the 3D volumetric rendering.

### Cephalometric analysis

2D and 3D cephalometric tracings were performed by two researchers with at least 10 years of experience in 2D and in 3D cephalometry (researcher I, CS; researcher II, GMT). These researchers repeated the tracings after 15 days to assess repeatability and reliability. Each patient was identified using a random identification code, and the researchers were blinded to the subjects’ identities when performing 2D and 3D cephalometric analysis.

Three reference planes for the 3D cephalometric analysis were identified as follows:

- Midsagittal plane (MSP) passing through Ba (Basion), S (Sella), and N (Nasion);
- Axial plane passing through N, S and normal to MSP;
- Coronal plane passing through S and normal to the other two planes.

Sella is intersected by the three anatomical planes, and it is the center of the reference system (point 0, 0, 0).

**Fig. 2:**
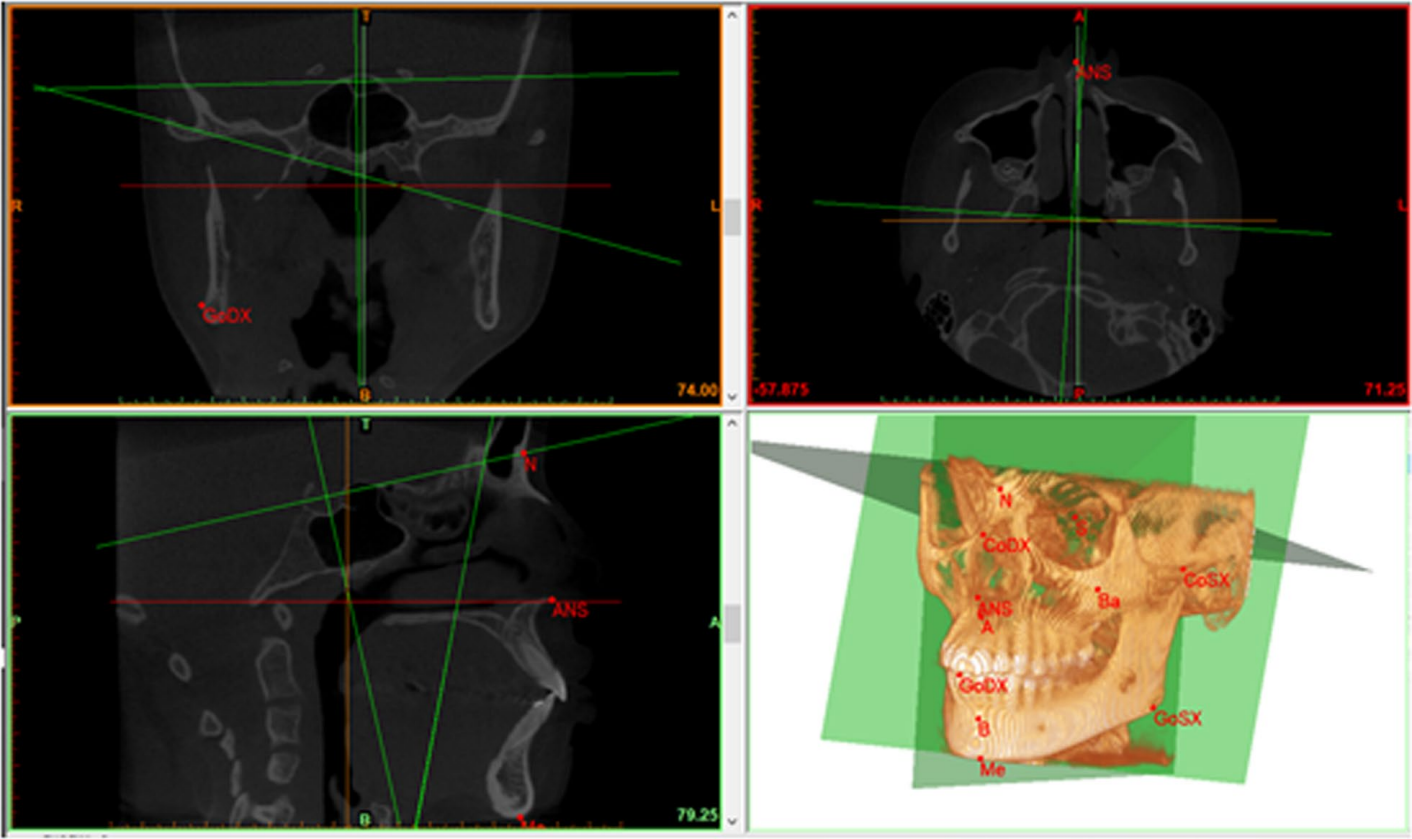
Cephalometric tracing of a CBCT on Materialise Mimics. At the bottom right some cephalometric points and all the three reference planes are visible

Fourteen cephalometric landmarks, ten unpaired (i.e., on the MSP) and four lateral symmetrical, were identified in CBCT axial, coronal, and sagittal sections: N (Nasion), S (Sella), Ba (Basion), A (Point A), B (Point B), Me (Menton), PNS (Posterior Nasal Spine), ANS (Anterior Nasal Spine), UI (Upper Incisor), LI (Lower Incisor); and paired landmarks: Sor (Supra Orbital), Mx (Maxillar), Co (Condylion) and Go (Gonion). The position of each point was then checked on the 3D volumetric rendering (Fig. 2).

The cephalometric analysis was performed according to classical Steiner methods [20]. A total of 15 measurements (20 considering the paired ones, too): 8 linear (unit: mm) of which 3 paired and 7 angular (unit: degrees) measurements in the vertical, sagittal, and transverse planes of which 2 paired were automatically generated by the program. Measurements taken into consideration are listed and explained in Table 1.

**Table 1:** List and description of cephalometric measurements used in this analysis

Linear measurements (mm)	PNS-A, Maxillary length: the distance between PNS and A	Unpaired
	S-N, Anterior cranial fossa length: the distance between S and N	Unpaired
	N-Me, Total anterior facial height: the distance between N and Me	Unpaired
	N-ANS, Upper anterior facial height: the distance between N and ANS	Unpaired
	ANS-Me, Lower anterior facial height: the distance between ANS and Me	Unpaired
	Go L/R-Me, Mandibular body length: the distance between left and right Go and Me	Bilateral
	Go L/R -S, Posterior facial height: the distance between S and left and right Go	Bilateral
	Go L/R-Co L/R, Mandibular ramus height: the distance between left and right Cd and Go	Bilateral
Angular measurements (deg)	SNA, Anteroposterior projection of the maxilla: the angle formed between points S, N, and A	Unpaired
	SNB, Anteroposterior projection of the mandible: the angle formed between points S, N, and B	Unpaired
	ANB, Anteroposterior intermaxillary relationship: the angle formed between points A, N, and B. In 3D analysis, unlike traditional cephalometrics, the difference between SNA and SNB could differ from the value of ANB	Unpaired
	BaŜN, Cranial base angle: the angle between Ba, S, and N	Unpaired
	S-N^PNS-ANS, Craniomaxillary angle: the angle between the floor of the anterior cranial fossa and the palatal plane	Unpaired
	PNS-ANS^Go R/L-Me, Maxillomandibular (intermaxillary) angles: the angles between the palatal and mandibular planes	Bilateral
	S-N^Go R/L-Me, Craniomandibular angle: the angle between the floor of the anterior cranial fossa and the mandibular plane, measuring mandibular divergence	Bilateral

### Statistical analysis

A preliminary analysis was performed on the records of 30 patients to gain information for sample size calculation. Sample size calculation was performed a priori using MedCalc® Statistical Software version 20.013 (MedCalc Software Ltd, Ostend, Belgium; https://www.medcalc.org; 2021) to estimate the required total number of cases for a method comparison study using the Bland–Altman statistics to obtain a statistical power of the study greater than 0.95 at an α = 0.05. The data used in the analysis were relative to PNS-ANS^Go L-Me as they were that required: Mean Difference between the two methods = 14.76, σ = 2.61 and Maximum Allowed Difference between methods equal to 21.52. Based on these parameters, the required sample size was 105 patients. This measurement was chosen as it was the one that required the largest sample size, thus ensuring a sufficient statistical power for all the variables under evaluation in the present study. In order to ensure robustness of data 114 records were selected.

Before further analyses, paired t-tests were conducted between left and right homologous measurement to check if they were significantly different. As shown in Table 2, there was not a significant difference between the two sides for all the considered measurements, so bilateral values were averaged. The reason of this choice was to reduce the number of variables, also considering that 2D cephalometric analysis was performed on 2D RLCs, thus on 2D projected images.

**Table 2:** Left and right homologous measurements and paired t-test between sides for each variable

Left and right-side measurements comparison						
Measurement	3D CBCT			2D RLC		
	Mean	SD	p-value [−]	Mean	SD	p-value [−]
Go L-Me [mm]	77.0	5.65	0.551	65.0	5.46	0.905
Go R-Me [mm]	77.4	5.66		64.9	5.80	
Go L-S [mm]	78.9	7.82	0.955	67.0	7.78	0.990
Go R-S [mm]	78.9	7.48		67.0	7.78	
Go L-Cd L [mm]	50.1	9.34	0.996	49.3	6.63	0.859
Go R-Cd R [mm]	50.1	8.87		49.4	6.56	
PNS-ANS^Go L-Me [deg]	41.6	3.23	0.713	26.8	4.53	0.892
PNS-ANS^Go R-Me [deg]	41.4	3.48		26.9	4.60	
S-N^Go L-Me [deg]	46.5	3.69	0.985	35.0	4.77	0.898
S-N^Go R-Me [deg]	46.5	3.99		35.1	4.98	

See Table 1 for abbreviations

The collected data were statistically analysed using IBM SPSS software (IBM Corp. Released 2020. IBM SPSS Statistics for Windows, Version 27.0. Armonk, NY: IBM Corp). Data distribution was assessed using Kolmogorov-Smirnoff test and confirmed data had a Gaussian distribution. In order to evaluate the intra-rater reliability, the variation of data measured by the same rater in two observations under the same conditions, an Intra Class Coefficient (ICC) was calculated. To quantify the inter-rater reliability, the variation of data measured by different raters, the ICC was estimated after a multilevel mixed-effects linear regression. The values of intra- and inter-rater ICCs were interpreted according to Cicchetti and Sparrow [21]: [0; 0.40) poor, [0.40; 0.60) fair, [0.60; 0.75) good, and [0.75; 1.0] excellent reliability. After this check, averaged values across all operators’ measurements were considered.

The mean value and standard deviation of each measurement were computed separately for 3D and 2D values. Standard Errors of Measurement (SEM) and 95% Confidence Intervals (CI) were calculated. Moreover, agreement and correlation between the two sets of values, 3D and 2D cephalometric measurements, were evaluated using Bland–Altman and scatter plots, respectively. In particular, Bland–Altman analysis evaluates the agreement between two sets of measurements, and it is usually used in a clinical context to compare a new measurement method against a gold standard. In this study, the averaged values associated to linear and angular measurements relative to each patient were employed for Bland–Altman analysis. The diagram was constructed by plotting the differences *d*_*i*_ between patient $i$ 3D vs 2D measurement value on the y-axis against their averaged value *m*_*i*_ on the x-axis.

$${d}_{i}= {3Dvalue}_{i}-{2Dvalue}_{i},$$

$${m}_{i}= \frac{{3Dvalue}_{i}+{2Dvalue}_{i}}{2},$$

$$i=1, \dots , number of patients.$$

with bias $\overline{d }$ between the two sets equal to the mean of the differences *d*_*i*_:

$$\overline{d }= \frac{{\sum }_{i}{d}_{i}}{number of patients}$$

and upper and lower levels of agreement ($LOA$) equal to

$$LOA= \overline{d }\pm 1.96*s$$

where *s* is the standard deviation across ${d}_{i}$ differences, and $LOA$ represent the limits at the 95% confidence interval of having normally distributed 3D vs 2D differences.

### Results

Table 2 reports the paired t-test between left and right homologous measurements. The test was performed for both 2D and 3D sets of values to check for differences between them. As shown in the table, homologous values were not significantly different for all the considered measurements (p-value > 0.05), so the averaged value between them was considered for each pair.

Table 3 reports the results of intra-rater and inter-rater reliability evaluated for each measurement. In all sets, the values of intra- and inter-rater ICC showed excellent repeatability and reliability for both 2D and 3D measurements (ICC > 0.75) [21]. The average ± standard deviation (SD) intraobserver ICCs were 0.98 (± 0.01) and 0.97(± 0.01), respectively for CBCT and 2D cephalograms. Inter-rater reliability resulted in an average ICC (± SD) of 0.98 (± 0.01) for CBCT and 0.94 (± 0.03) for 2D cephalometry.

**Table 3:** Reliability and repeatability of the two different cephalometric tracings assessed through inter operator and intra operator ICC

Reliability				
Measurement	3D CBCT		2D RLC	
	Intraobserver ICC [–]	Interobserver ICC [–]	Intraobserver ICC [–]	Interobserver ICC [–]
PNS-A	0.99	0.97	0.98	0.96
S-N	0.97	0.96	0.96	0.84
N-Me	0.99	0.97	0.98	0.95
N-ANS	0.99	0.97	0.96	0.95
ANS-Me	0.98	0.98	0.98	0.96
Go-Me	0.98	0.96	0.97	0.94
Go-S	0.99	0.98	0.98	0.96
Go-Cd	0.98	0.97	0.97	0.93
SNA	0.97	0.95	0.96	0.92
SNB	0.95	0.92	0.93	0.91
ANB	0.99	0.97	0.96	0.94
BaŜN	0.99	0.97	0.97	0.94
S-N^PNS-ANS	0.98	0.97	0.96	0.93
PNS-ANS^Go-Me	0.99	0.98	0.98	0.95
S-N^Go-Me	0.99	0.97	0.97	0.94

See Table 1 for abbreviations

**Fig. 3:**
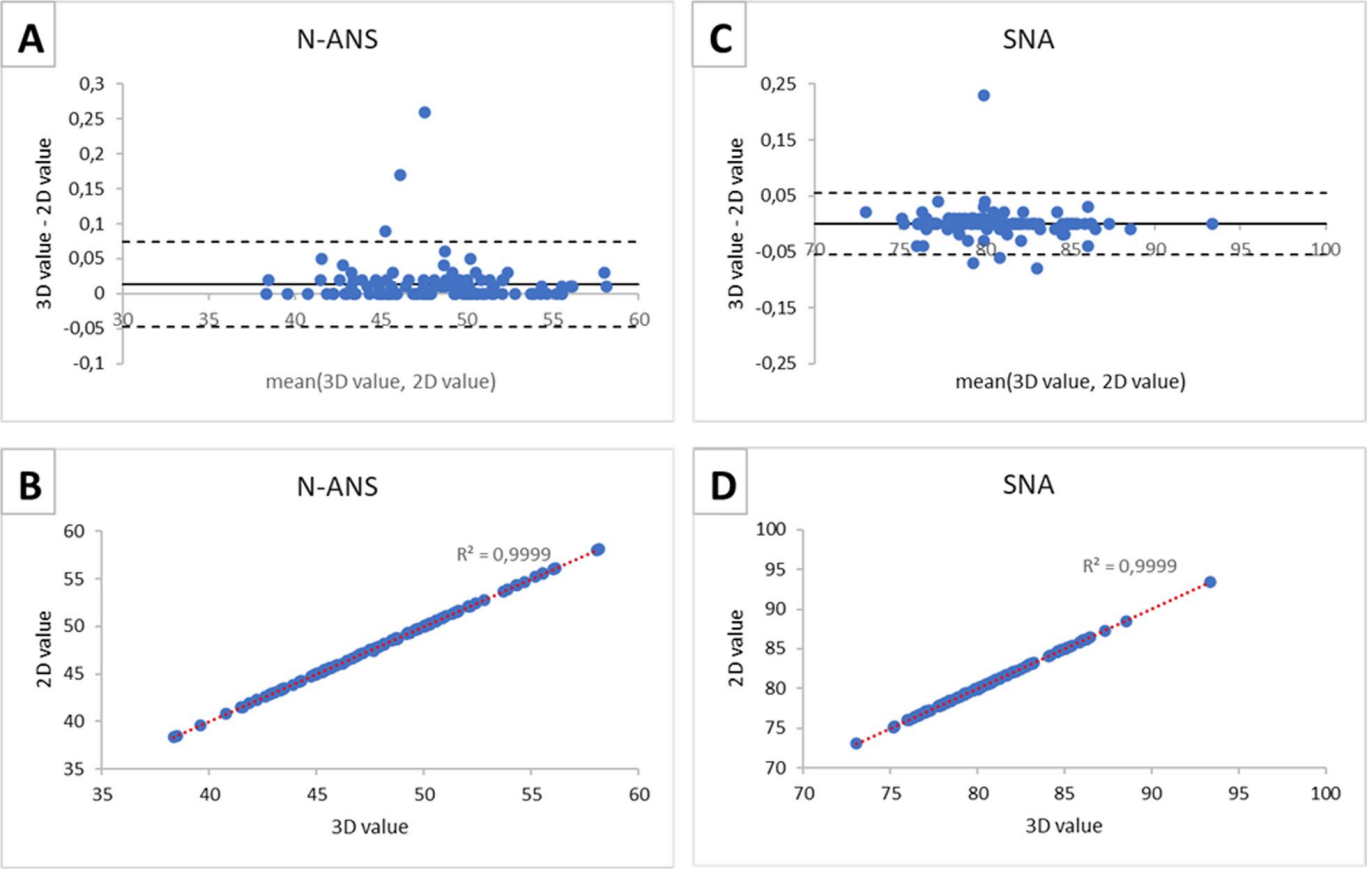
Bland–Altman plots (panels **A**, **C**) and scatter plots (panels **B**, **D**) between CBCT measurements (3D values) and RLC measurements (2D values). Results are presented for unpaired measurements: linear measurement N-ANS and angular measurement SNA

**Fig. 4:**
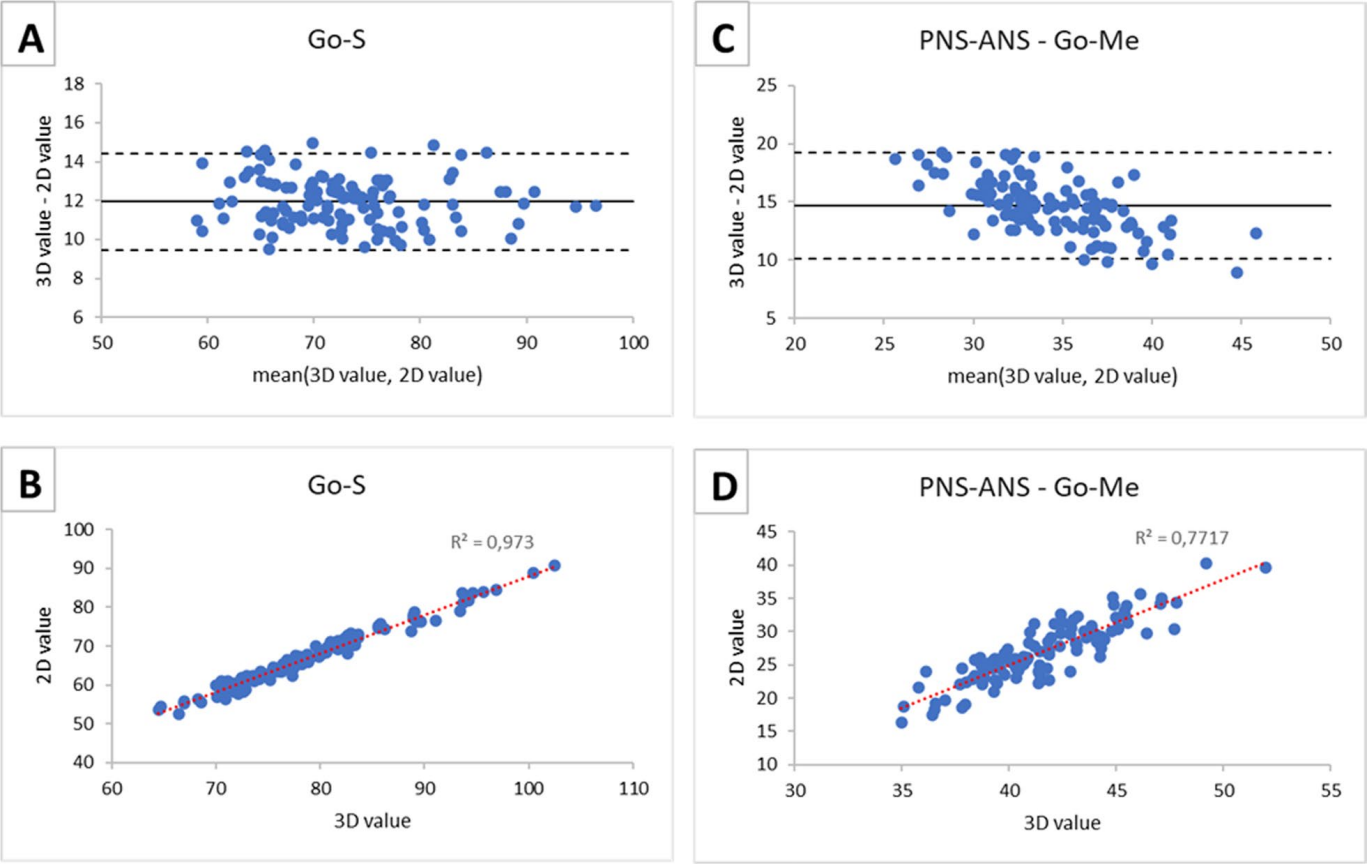
Bland–Altman plots (panels **A**, **C**) and scatter plots (panels **B**, **D**) between CBCT measurements (3D values) and RLC measurements (2D values). Results are presented for bilateral measurements: linear measurement Go-S and angular measurement PNS-ANS^Go-Me

Table 4 presents mean, SD, SEM, lower and upper limits of 95% CIs of cephalometric values obtained using the 3D and 2D analyses and paired t-test between 2 and 3D measurements. Table 5 presents the comparisons by means of correlation coefficients and Bland–Altman analysis for the same variables. In general, all the sets of measurements showed strong positive correlations: 0.995 ± 0.012 for unpaired measurements and 0.924 ± 0.060 for bilateral measurements. For Bland–Altman analysis, the biases and ranges for the 95% Limits Of Agreement of each measurement showed higher levels of agreement between the two modalities for unpaired measurements relative to bilateral ones. For unpaired measurements, bias was in the range [0.01; 0.02] mm for linear and [− 0.02; 0.13]° for angular values, while for bilateral measurements bias was between 0.27 and 12.30 mm for linear and between 11.51 and 14.64° for angular values. Since the Bland Altman plots and the scatter plots relative to the measurements that belong to the same group (i.e. bilateral and unpaired measurements) show similar trends, one linear variable and one angular variable were selected for each group so as to avoid redundancy. The Bland Altman plots and the scatter plots relative to unpaired measurements (N-ANS and SNA) are shown in Fig. 3 and those relative to bilateral measurements (Go-S and PNS-ANS^Go-Me) are reported in Fig. 4.

**Table 4:** Descriptive statistics (mean, SD, SEM), lower and upper limits of 95% CIs and paired t test between measurements performed on CBCT and on reconstructed lateral cephalograms

Descriptive statistics, confidence intervals and paired t tests											
Measurement	3D CBCT			CI		2D RLC			CI		p-value [–]
	Mean	SD	SEM^a^	Lower	Upper	Mean	SD	SEM^a^	Lower	Upper	
PNS-A [mm]	44.0	3.38	0.32	43.33	44.57	43.9	3.38	0.32	43.32	44.56	0.976
S-N [mm]	64.8	3.94	0.37	64.07	65.51	64.8	3.94	0.37	64.06	65.50	0.986
N-Me [mm]	105.7	8.90	0.83	104.10	107.37	105.7	8.89	0.83	104.08	107.35	0.986
N-ANS [mm]	48.0	4.14	0.39	47.24	48.76	48.0	4.14	0.39	47.23	48.75	0.980
ANS-Me [mm]	58.8	5.76	0.54	57.78	59.90	58.8	5.76	0.54	57.76	59.87	0.977
Go-Me[mm]	77.2	5.54	0.52	76.20	78.23	64.9	5.48	0.51	63.91	65.92	< 0.001
Go-S [mm]	78.9	7.61	0.71	77.52	80.32	67.0	7.71	0.72	65.55	68.38	< 0.001
Go-Cd [mm]	50.1	9.04	0.85	48.43	51.75	49.4	6.51	0.61	48.16	50.54	0.744
SNA [deg]	80.5	3.29	0.31	79.89	81.10	80.5	3.29	0.31	79.89	81.10	1.000
SNB [deg]	78.2	3.11	0.29	77.60	78.74	78.2	3.12	0.29	77.59	78.74	0.988
ANB [deg]	2.5	1.07	0.10	2.33	2.72	2.4	1.09	0.10	2.20	2.60	0.371
BaŜN [deg]	129.9	5.58	0.52	128.83	130.87	129.9	5.58	0.52	128.85	130.90	0.977
S-N^PNS-ANS [deg]	8.3	3.13	0.29	7.72	8.87	8.2	3.19	0.30	7.59	8.77	0.787
PNS-ANS^Go-Me [deg]	41.5	3.07	0.29	40.94	42.07	26.9	4.49	0.42	26.04	27.69	< 0.001
S-N^Go-Me [deg]	46.5	3.68	0.34	45.86	47.21	35.0	4.81	0.45	34.14	35.90	< 0.001

^a^ SEM, Standard Error Measurement; See Table 1 for abbreviations

**Table 5:** Correlation coefficient and Bland–Altman analysis

3D CBCT–2D RLC comparison					
Measurement	Correlation coefficient [–]	Bias	Lower LOA	Upper LOA	LOAs range
PNS-A [mm]	0.999	0.01	− 0.05	0.07	0.12
S-N [mm]	0.999	0.01	− 0.03	0.05	0.07
N-Me [mm]	0.999	0.02	− 0.08	0.12	0.19
N-ANS [mm]	0.999	0.01	− 0.05	0.07	0.12
ANS-Me [mm]	0.999	0.02	− 0.06	0.11	0.17
Go-Me [mm]	0.966	12.30	9.48	15.12	5.64
Go-S [mm]	0.986	11.96	9.48	14.44	4.96
Go-Cd [mm]	0.844	0.27	− 0.17	0.72	0.89
SNA [deg]	0.999	0.00	− 0.05	0.05	0.11
SNB [deg]	0.999	0.00	− 0.05	0.05	0.11
ANB [deg]	0.960	0.13	− 0.47	0.73	1.19
BaŜN [deg]	0.999	− 0.02	− 0.13	0.08	0.21
S-N^PNS-ANS [deg]	0.998	0.11	− 0.31	0.54	0.84
PNS-ANS^Go-Me [deg]	0.878	14.64	10.10	19.18	9.08
S-N^Go-Me [deg]	0.944	11.51	7.98	15.04	7.06

See Table 1 for abbreviations

Scatterplots (panels B and D of Figs. 3 and 4) showed an overall linear correlation between 3 and 2D measurements across all conditions. Unpaired measurements showed better agreement in the Bland–Altman plots and higher correlation values in the scatterplots compared to bilateral measurements (see Table 5).

## Discussion

While most of the clinical orthodontic knowledge related to craniofacial analysis derives from 2D cephalometric analysis, the assessment of linear and angular measurements using 2D radiographs raises an important issue as a 3D object is flattened into a 2D image. In fact, the main disadvantages of the 2D conventional cephalometric analysis are represented by projective distortions and rotational errors, which might affect the reliability and reproducibility of the measured values, in particular those not belonging to the MSP [3]. In order to overcome the limitations associated with traditional cephalometric analysis, cephalometric measurements using CBCT images have been introduced, and found to have a similar performance than 2D cephalometric tracings, with the great advantage to provide reliable 3D information while using a single cephalometric analysis and not two projections (i.e., lateral and posteroanterior) [22, 23]. To date, CBCT imaging has a crucial role for the assessment and management of complex cases in orthodontics and in maxillofacial surgery. Since it delivers higher radiation doses compared to 2D radiology, its application is limited to specific cases where a 3D assessment of the jaw is required. In these cases, performing 3D cephalometry directly on the CBCT scan could be useful to avoid delivering further radiation.

The current study pointed out the differences in measuring distances and angles that are coplanar and not to the MSP using CBCT and RLCs with the aim to identify which cephalometric measurements can be measured by both techniques with an acceptable margin of error. To limit the biological variety and focus on inter subject variability, in this first study only symmetrical Class I patients were selected; the paired t-test between sides confirmed the absences of statistically significant differences for each paired measurement and allowed to consider the averaged values between sides in further analysis. The paired t-tests between CBCT and RLC revealed that the values whose differences were statistically significant were Go-Me, Go-S, PNS-ANS^Go-Me and S-N^Go-Me. These results can be explained by the distortion error due to the lateral landmarks projection on the MSP. About that, a recent article compared RLC and CBCT [14] focusing on the changes between imaging techniques’ outcomes during mandibular growth and reported that the extent of distortion was positively correlated to the angle of incidence of the segment Go-Me. In our study, Go-Me and Go-S were found to suffer a similar distortion for the same reason. Considering the two angular measurements (PNS-ANS^Go-Me and S-N^Go-Me), the statistically significant difference between the methods can be due to the interaction of a line that is coplanar with the MSP and a segment that is instead para-axial to it.

Scatterplots demonstrated the high positive correlation between 2 and 3D measurements, while further information could be obtained by Bland–Altman analysis, the last providing useful information to assess the bias of the measurements to formulate educated guesses about their correction using specific algorithms to be investigated in future studies. For all unpaired measurements the bias was close to 0, meaning that there was not a systematic error between the two sets of 3D and 2D measures. Moreover, the limits of agreement were narrow and there were few outliers with respect to them: the two methods were essentially equivalent. On the other hand, even if the correlation between bilateral measurements obtained by 2D and 3D techniques was strong, Bland–Altman analysis evidenced a bias between the two sets. This bias was not a constant value for all the considered patients and values were normally distributed inside the LOAs. An exception was represented by the linear measurement Go-Co: the difference between the averages of CBCT and RLCs measurements was not significant (p > 0.05), the bias was close to zero (0.27 mm) and the LOAs were quite narrow (-0.17; 0.72 mm).

This was allegedly due to anatomical reasons: this linear measurement (mandibular ramus height) bears almost no distortion when projected into the MSP since it is almost parallel to it. Similar results were previously obtained for the same measurements although with different methods [18]. In general, for angular measurements, such as PNS-ANS^Go-Me in Fig. 4, there was a trend in the distribution of the differences against the mean values, i.e., differences between the methods tend to get smaller as the mean increases. Probably, for smaller angles, the error of projection on the MSP causes a relatively greater projective error. Furthermore, high angle patients usually present reduced mandibular width [24] thus a reduced distortion as the Go-Me segment is less rotated from the MSP. The lowest correlation between 2 and 3D measurements was relative to PNS-ANS^Go-Me. This event could allegedly be due to the unpredictable distortion of the segment Go-Me that affects the angle in an unpredictable way since the other segment of this angle is not coplanar with it and belongs to the MSP.

Both techniques appeared to have excellent reproducibility (0.98–0.96 for CBCT and 0.96–0.84 for RLC) and reliability (0.99–0.97 for CBCT and 0.98–0.96 for RLC) as measured by ICC index and therefore they appeared allegedly comparable in terms of diagnostic accuracy. Available literature agrees on the high reproducibility and reliability of both the imaging methods as assessed in studies using physical measurements and scans made on dry skulls [3, 17, 25, 26] and in other studies comparing cephalometric measurements on 2D cephalograms and on CBCT scans [13, 27, 28], but little information on which measurements are subjected to greater errors and which of them can be corrected and efficiently used for orthodontic diagnosis are evaluated [18, 29].

To the authors’ knowledge, in the first study that applied Bland–Altman analysis to assess biases between conventional 2D measurement system and 3D imaging method [3], dry skulls were measured using a digital caliper to establish the linear physical measurements as a gold standard to make comparisons between lateral cephalograms and reconstructed 3D images. However, the 3D imaging relied only on the acquisition of three 2D cephalograms (standard lateral (90°), frontal (0°), and oblique (45°)) merged into the same 3D matrix. The evaluated 3D approach showed high precision with a greatly reduced bias to the gold standard and much less variability in its measure compared with the conventional 2D approach.

Regarding the evaluation of the difference between conventional 2D cephalometry and CBCT in vivo on patients, Oz et al. [13] performed a study on 11 patients with several limitations: despite sample size calculation was declared no information on the exact calculation was provided nor allegedly sufficient sample numerosity was reached to obtain meaningful inferential statistics. Also, no clear description of 3D cephalometric measurements was given and no information regarding bias between the two imaging methods was given. They found no statistical differences among 2D and 3D CBCT-generated cephalogram measurements, except for Go-Me and Condylion-Gnathion (Co-Gn) linear measurements. Li et al. [28] analyzed the differences between CBCT and RLC cephalometric methods on 40 patients by means of a paired *t*-test. The results indicated that the two methods showed significant differences in all measurements (SNA, SNB, ANB, MP-FH, SND, U1-NA linear mesurement, U1-NA angle, U1-L1, L1-NB linear measurement, L1-NB angle, L1-MP, L1-FH, OP to SN, Pog to NB, GoGn to SN). These results however are mainly due to the statistics that have been chosen to perform the comparison. In fact a paired t-test detects systematic differences even if they are of low or of no clinical significance. Pittayapat et al. [27] focused on the comparison of the CBCT vs physical measurements and lateral cephalometry vs physical measurements measured on dry skulls by means of inter- and intra-observer variability expressed as a percentage of coefficients of variability (absolute difference between methods below 1%). No direct comparison was performed between CBCT and lateral cephalogram nor any inferential statistics aiming to compare coefficients of variation, as done in other studies [14], was performed. The Go-Me segment was the most affected by the distortion of the projection, as found in our study. Gribel et al. [18] performed 12 craniometric measurements on 25 dry skulls and compared them with cephalometric indexes on lateral teleradiography and on CBCT scans. No meaningful difference was observed between the direct assessments on dry skulls and the on CBCT scans on analysis of variance (ANOVA) (P > 0.05). The comparison between all the cephalometric indexes and the craniometrics measurements retrieved statistically significant results (Tukey test, P < 0.05). Great differences were observed between different variables. Gribel et al. [29] stated that none of assessed cephalometric variables measured in CBCT were significantly different from the craniometrics assessments if a trigonometric algorithm correction was applied on 2D measurements taken on lateral cephalogram. However, its evidence has low clinical significance since the CBCT data were used to derive individualized correction factors that are applicable for that particular subject only. Measurements on the MSP were calculated simply by reducing the 10% magnification of the cephalometric distortion since the MSP is already parallel to the cephalometric film. However, in all these studies no information regarding bias between the two imaging methods was given.

### Limitations of the study

In this study, patients with facial asymmetry in the bilateral cephalometric variables greater than 2 mm were excluded to reduce the errors of 2D RLC tracing in order to obtain a simplified model to test our hypothesis that measurements not laying on the MSP are affected by a certain amount of distortion that could be estimated and corrected.

Also, the study investigates only skeletal Class I patients; further investigations should focus on the morphological cases that occur on skeletal Class 2 and 3 patients. For these patients with more complex morphologies, a CBCT is usually available to assess the facial structure and investigate the asymmetry between the sides of the face. However, the study design would be much more complex. Moreover, a possible development of the present study could include the estimation of 2D measurements from 3D ones. Measuring 2D from 3D linear and angular distances can be useful in cases where you have a patient's CBCT and want to trace 2D data without exposing the patient to further radiation.

The analysis of the effect of the facial orthopaedic treatment of the mandibular shape could also be of some interest. Evaluation of patients with different skeletal classes and those with varying degrees of asymmetry would be useful. Such data would allow conclusions to be drawn that could be more easily extended to the entire population, since the results of the present study only fit people with a satisfactory degree of facial symmetry and a normal anteroposterior maxilla-mandibular relationship. It may be useful to widen the sample size by a multicentre approach so to increase the robustness of our findings on this debated topic.

## Conclusion

It appears that most cephalometric indexes investigated in this study can be measured on CBCT and used for orthodontic diagnosis since they do not present statistically significant differences with the ones measured on 2D lateral cephalograms. However, the measurements where one or more landmarks lay far from the MSP bear distortions that could allegedly be overcome using specific formulas converting 3D values into 2D ones as the performed Bland–Altman and correlation analyses seem to suggest. The future development of specific algorithms for this purpose could help clinicians avoid exposing patients to unnecessary 2D cephalometric lateral radiographs where CBCT is indicated and to deduct all information needed for orthodontic diagnosis from the CBCT scan.

## Data Availability

The data analysed during the current study are available on request due to restrictions, e.g., privacy or ethical.

## Abbreviations

CBCT: Cone beam computed tomography
3D: Three-dimensional
2D: Two-dimensional
RLC: Reconstructed lateral cephalogram
A Point: A of Down’s
B Point: B of Down’s
ANS: Anterior nasal spine point
PNS: Posterior nasal spine point
Me: Menton point
Ba: Basion point
S: Sella point
Co: Condylion point
Go: Gonion point
UI: Upper incisor point
LI: Lower incisor point
Sor: Supra orbital point
Mx: Maxillary point
ICC: Intraclass correlation coefficient
MSCT: Multi slice computed tomography
MSP: Midsagittal plane
N: Nasion point
CI: Confidence interval
SEM: Standard errors of measurement
LOA: Levels of agreement
